# Comparative Evaluation of *SMAD-2* Expression in Oral Submucous Fibrosis and Reactive Oral Lesions

**DOI:** 10.31557/APJCP.2020.21.2.399

**Published:** 2020

**Authors:** Sravya Zagabathina, Ramya Ramadoss, Harini Priya AH, Rajkumar Krishnan

**Affiliations:** 1 *Department of Oral Pathology and Microbiology, SRM Dental College, SRM University, *; 2 *Department of Oral Pathology, Chettinad Dental College and Research Institute, Chennai, India. *

**Keywords:** SMAD, OSMF, Reactive oral lesions

## Abstract

**Background:**

The event of fibrosis encompasses involvement of definite immunological and molecular mechanisms. As quite a lot of pro-fibrotic pathways are concerned, a multipronged approach is obligatory to cognize the fibrotic events. SMAD signaling pathway hasn’t been studied oral fibrotic events.In the progression of cramming the SMAD signaling pathway in OSMF, the first initiator protein of the pathway was considered for evaluation in the present study.

**Materials and Methods:**

A total of 100 subjects consisting of 20 controls, 40 patients with reactive lesions such as Traumatic Fibroma, Epulis Fissuratum and Gingival Hyperplasia and 40 patients with Oral Submucous Fibrosis were recruited for the study. Tissue homogenates were assayed by quantitative sandwich enzyme immunoassay technique using Human Mothers Against Decapentaplegic Homolog 2 (Smad2).

**Results:**

*SMAD 2* expression values showed significant difference between control and OSMF group. However, the difference between reactive lesions with control and OSMF were not statistically significant.

**Conclusion:**

Graded increase of *SMAD 2* expression from control,reactive lesions and OSMF were observed accentuating the role of SMAD signalling pathway in fibro genesis. Further this can be validated to generate effective antifibrotic targets.

## Introduction

Oral sub mucous fibrosis (OSMF) is a chronic progressive premalignant disorder. Clinical manifestations encompass bigotry to spicy food, stringency of lip, tongue and palate leading to varying degrees of constraint of opening of the mouth and tongue movement (Ekanayaka and Tilakaratne, 2016). The disease is witnessed in countries where the habit of betel chewing is recurrently practiced. The rate of malignant transformation is 7-13 % (Jayasinghe et al., 2016). Albeit the available epidemiological evidence implies that the chewing of areca nut is a definitive risk factor for progress of OSMF, imperceptibly chewers procure the disease and it is not a dose–response related disease. Desuetude of the habit also does not impact the characteristics of the disease once it is ensued. Regardless of copious recent advances in the molecular biology of the events liable for the regulation of genes encoding collagens and other extra cellular matrix proteins, there is curbed knowledge regarding the profound mechanisms behind irreversible fibrosis. Discordant to other fibrotic disorders, research in OSMF is limited. Treatment available so far is based on a symptomatic approach.

In general, the event of fibrosis encompasses involvement of definite immunological and molecular mechanisms. Modified innate and adaptive immune responses are major contributors to fibrosis (Wick et al., 2010). As quite a lot of pro-fibrotic pathways are concerned, a multipronged approach is obligatory to cognize the fibrotic events. In depth analysis of the fibrotic events for conception of ideal anti-fibrotic therapy for OSMF is the desideratum of the hour. Transforming growth factor β (TGF-β) is deliberated to be a potent stimulator of development and deposition of extra cellular Matrix (Rajalalitha and Valli, 2005), which also plays a critical role in embryonic development, immune responses, and regulation of tissue repair subsequent to injury (Rosenbloom and Jimenez, 2008).

The delimitation of the acute role of TGF-β in the development of embellished tissue fibrosis, and the identification of the unambiguous cellular receptors, kinases and intracellular mediators which participate in the cellular response to TGF-β in the initial stages of tissue fibrosis, is most substantial than any other molecules as these TGF-β give the impression to be contemporaneous in amplified levels in OSMF paralleled to that of normal Tissue (Rosenbloom and Jimenez, 2008). Malformations in signaling of TGF-β leads to serious human diseases like cancer, fibrosis, wound healing disorders (Schmierer et al., 2007).

TGF-β signal transduction from membrane into the cell nucleus involves two functional TGF-β receptors and SMAD (Small Mother against Decapentaplegic homolog) family of proteins which is on the nether side of scrupulous regulation (Lo and Massague, 1999).

SMAD is a group of transcription factors that officialize as signal transducers of TGF-β and for sub sequential response by direct regulation of gene expression. Though SMAD signaling is sluggish in its onset, it is exceedingly unrelenting one time instigated (Schmierer et al., 2007).

SMAD 2 protein is a receptor regulated type of SMAD and intercedes the signal of the transforming growth factor (TGF)-beta. It integrates multiple cellular processes, such as cell proliferation, apoptosis, and differentiation. This protein is conscripted to the TGF-β receptors over and done with its interface with the SMAD anchor for receptor activation (SARA) protein (Joachim et al., 2018).

SMAD 2 turn into the instigating event in the complex TGF-β pathway. Literature exploration publicized a single institutional study on *SMAD 2* and *4* expressions in OSMF archived tissue samples by immunohistochemistry (Moutasim et al., 2011).

Complete SMAD signaling pathway hasn’t been studied in OSMF tissues hitherto. Hence in the progression of cramming the SMAD signaling pathway in OSMF, the first initiator protein of the pathway was considered for evaluation in the present study. The present study anticipates to scrutinize *SMAD 2* expression in tissue lysates of OSMF by Enzyme linked immuno sorbent assay (ELISA) with parallel evaluation in healthy controls and reactive lesions. Tissue lysates are further expedient in analysis of Proteins as the signals of weakly-expressed proteins too are straightforwardly evident.

## Materials and Methods

*Recruitment of Subjects*

The study was approved by the ethical committee of SRM university. Informed consent was attained from the patients or the relatives before entering into the study. A total of 100 subjects were conscripted for the study, 20 controls devoid of any systemic illness (12-male and 8-female with the mean age of 28±7.80), 40 patients with reactive lesions such as Traumatic Fibroma, Epulis Fissuratum and Gingival Hyperplasia (18-Male and 22-Female with a mean age of 32.55±14.8) and 40 patients with Oral Submucous Fibrosis (36-Male and 4-Female with a mean age of 36.95±7.9). Recruitment was based inclusion and exclusion criteria as mentioned in Table 1, 2 and 3.

*Tissue samples*

Tissues were sourced from all the 3 groups after obtaining due consent. Approximately 5 – 7 mm of tissue was taken under local anaesthesia.Gingival tissue excised during Crown Lengthening procedure was utilized for Group I subjects. All specimens were immediately placed in Eppendorf tubes and frozen. Samples were stored at -80^o^C freezer.

*Elisa Assay*

Tissue Homogenisation was done by rinsing the tissue with Phosphate Buffer Solution and stored overnight at -20°C. Two freeze-thaw cycles were performed to break the cell membranes. The tissue homogenates were centrifuged for 5 minutes at 5,000 rpm, at 2 - 8°C. The lysate was removed and assayed immediately using Human Mothers Against Decapentaplegic Homolog 2 (Smad 2) ELISA Assay kit (My Biosource, SanDiego, US). 100μl of tissue supernatant was added per well. 100μl of Biotin-antibody was added and incubated for 1 hour at 37°C. Wash buffer (200 μl) was used for three washes with a multi-channel pipette. Further 100μl of HRP-avidin was added and incubated for 1 hour at 37°C. 90μl of TMB Substrate was added to each well and incubated for 15-30 minutes at 37°C.50μl of Stop Solution to each well was added to inactivate the enzyme. Optical density of each well was read using a spectrophotometer using 450nm as the primary wave length.

*Statistical Analysis*

Comparison of the levels of SMAD 2 between groups was carried out using ANOVA followed by Tukey post hoc test. Statistical analysis of the data was obtained using SPSS software version 22.

## Results

The present study encompassed a total of 100 subjects, out of which 20 were Healthy controls, 40 were reactive lesions and 40 cases of Oral sub mucous fibrosis. Tissue lysates obtained from all three groups were investigated for expression of *SMAD-2* using quantitative sandwich enzyme immunoassay technique. Comparison of *SMAD-2* expression among all three groups were done. Comparison was also done for *SMAD -2* expression in 4 different clinical stages of OSMF and the types of reactive oral lesions.

The descriptive statistics such as mean and standard deviations [SD] were calculated for the individual groups and comparison of *SMAD 2* expression with in the three groups and clinical staging of OSMF using One- way ANOVA and tabulated as in Table 2. The comparison between the control group and the oral sub mucous fibrosis revealed there is significant difference between the values at the level of 95% (p value < 0.05).The comparison between the control group and reactive lesions group revealed that there is no significant difference between the values at the level of 95% (p value >0.05).The comparison between the reactive lesions and the oral submucous fibrosis revealed that there is no Significant difference between the values at the level of 95% (p value >0.05). *SMAD 2* expression values showed progressive increase from healthy controls to reactive lesions and OSMF.

Comparison of *SMAD2* expression among the various types of clinical staging of OSMF revealed a graded increase from stage I to IV. Similar observations were also found amidst histopathological grades. *SMAD 2* expression was found to be increased in inflammatory gingival hyperplasia than traumatic fibroma and epulis fissuratum.

**Figure 1:**
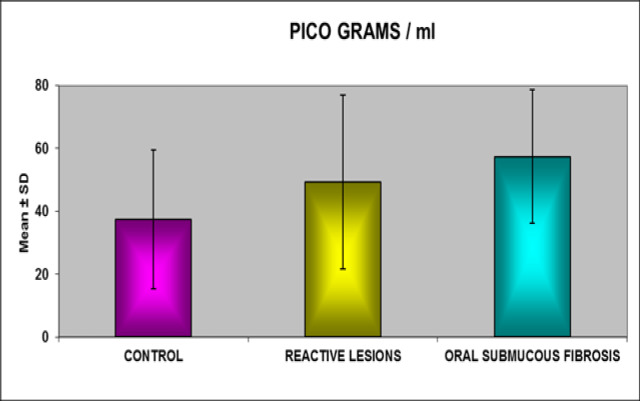
Comparison of SMAD-2 between Groups

**Table.1:** Inclusion and Exclusion Criteria for Samples

Group	Inclusion Criteria	Exclusion Criteria
Group I (Control)	Age and sex matched individuals with no systemic illness.	Individuals with systemic illness.
Group II (Reactive Lesion)	Diagnosed cases of Reactive lesions.	Individuals with systemic illness.
Group III (Oral Submucous Fibrosis)	Clinically diagnosed cases of Oral submucous fibrosis, based on Khana and Andrade ( 1995) Clinical staging. Histopathological confirmation was done using light microscopy and grading based on Pindborg and Sirsat ( 1966)	Patients who are currently undergoing or having undergone any form of treatment for Oral Submucous fibrosis.

**Table 2:** Demographic Details of Patients with Oral Submucous Fibrosis

Characteries		
Gender	Male	36
	Female	4
Age	20-29	6
	30-39	20
	40-49	8
	50-59	6
Clinical staging	I	4
Khana & Andrade	II	23
	III	10
	IVa	3
	IVb	-
Histopathological grading	Very early	-
Pindborg & Sirsat	Early	4
	Moderately advanced	27
	Advanced	9

**Table 3:** Demographic Details of Patients with Reactive Oral Lesions

Traumatic Fibroma ( n= 14)		
Gender	Male	6
	Female	8
Age	20-29	5
	30-39	7
	40-49	2
	50-59	-
Epulis Fissuratum ( n=12)		
Gender	Male	5
	Female	7
Age	20-29	-
	30-39	-
	40-49	4
	50-59	8
Inflammatory Gingival Hyperplasia (N=12)		
Gender	Male	4
	Female	8
Age	20-29	7
	30-39	4
	40-49	1
	50-59	-

**Table 4:** Comparison of SMAD -2 Mean Values between Healthy Controls, Reactive Lesions, and Oral Sub Mucous Fibrosis Groups

Group	Mean (pg/ml)	Standard Deviation	‘p’ Value	Level of Significance
Control	37.4175	22.16419		
Reactive lesions	49.2875	27.73846	0.267	Non-significant
Control	37.4175	22.16419		
OSMF	57.4200	21.29803	0.028	Significant at 95%
Reactive lesions	49.2875	27.73846		
OSMF	57.4200	21.29803	0.533	Non- Significant

p<0.05*

## Discussion

OSMF has the prospective to progress into invasive carcinoma and is termed as a premalignant disorder (Sabharwal et al., 2013). It has the highest rate of malignant transformation estimated to be 7-13% (Ekanayaka and Tilakaratne, 2016). Oral Squamous Cell Carcinoma originating from OSMF is clinically more intrusive and also unveils higher metastasis and recurrence rate than the OSCC not instigating from OSMF (Guo et al., 2011). Treatment modalities existing for OSMF is symptomatic and complete reversal of the disease has not been achieved till date. This is principally due to multifactorial aetiology and intricacy in pathogenesis. The main pathological modification in OSMF is amplified accretion of type I collagen confined by the sub-epithelial tissues. This is alleged to be a result from a disproportion between matrix deposition and dilapidation. Irreversible fibrosis is the ultimate aftermath of the disease. Fibrogenic cytokines such as Fibroblast growth factor, Plasma derived growth factor and transforming growth factor are imperative contributors of fibrosis. Many studies in the literature have proved the contribution of fibro genic cytokines (Kendall and Feghali., 2014).

Among the fibrogenic cytokines TGF β is well thought-out to be a persuasive stimulator of production and deposition of ECM. TGF β is the foremost trigger for the amplified collagen production and dwindled matrix degradation pathways in OSMF. Transforming growth factor β (TGF – β) superfamily entails of a diverse range of proteins that normalize many different physiological processes, including embryonic development, homeostasis, wound-healing, chemotaxis, and cell cycle control. TGF-β is triggered or unconstrained from complexes before binding to its cell-surface receptors, and intracellular signaling is promulgated to the nucleus through SMADS, mitogen- and stress-activated protein kinases and other pathways. Many of these steps are reformed in diseases, and each has the prospective to be targeted in treatment. Activation/phosphorylation of the SMAD complex has been concerned in the fibrotic events in diseases like hypertensive nephropathy, pulmonary fibrosis, hepatic fibrosis, diabetic nephropathy, systemic sclerosis and keloids. Targeting the SMAD complex has been successfully tried in the many of the above cited diseases. 

SMAD signalling pathway hasn’t been studied in OSMF tissues hitherto. Only one study conducted by Moutasim et al 2011, with 2 proteins of SMAD complex -SMAD 2 and 4 have been studied so far. Hence in the process of studying the SMAD signalling pathway in OSMF, the first spur protein of the pathway - SMAD 2 was deliberated for appraisal in the present study. 

Comparative analysis of expression of *SMAD 2* in all the three groups publicised progressive upsurge from healthy controls, reactive lesions and OSMF. Expression levels concerning Healthy control and OSMF group was statistically significant. There was no significance between the expression levels of healthy control and reactive lesions. Expression levels amid reactive lesions and OSMF also be deficient in significant difference.

Echelons of expression between the diverse clinical stages of OSMF was not statistically significant, however progressive increase was noted with clinical stages and histopathological grades. This fact could be indorsed to the extensive fibrosis in the metier of the protein expression.

Expression of *SMAD 2* in healthy controls were in accord with Dagmarapiestrzeniewicz et al 2003, who studied the expression of *SMAD 2* in typical endometrial tissue in contrast with neoplastic endometrial tissue. Increased expression of *SMAD2* in reactive oral lesions as well as oral submucous fibrosis is in agreement with a study conducted by Moutasim et al., (2011), who also reported amplified expression of* SMAD 2*, *4* in archived tissue specimens of fibroepithelial hyperplasia. Our study also showed significant increase in inflammatory gingival hyperplasia than traumatic fibroma and epulis fissuratum. This finding could be attributed to the key events mediating fibrosis in inflammatory gingival hyperplasia. There is a prominent role of inflammation in inflammatory gingival hyperplasia when compared to the other reactive lesion included in the study leading to increased fibrotic activity.

Phosphorylation of SMAD 2 was observed by Khan et al., (2012), subsequent to treatment of epithelial cells by catechin, tannin and alkaloids, suggesting areca nut interceded activation of p-SMAD 2. However, the advanced proliferation in expression of *SMAD 2* is an imperative finding which accentuates its role in the pathogenesis of OSMF. Evidence of TGF-β signalling as the chief causative event for amplified collagen production in OSMF has been corroborated in many studies. The present study reiterates the role of TGF-β in OSMF as SMAD 2 is the initiator molecule for the intracellular transcription of TGF-β.

In conclusion, the presence of SMAD 2 was found in all the three study groups and demonstrated a progressive increase from healthy controls, reactive lesions and OSMF, While OSMF disclosed the utmost expression of all. The present study thus reiterates the role of TGF-β in OSMF as SMAD2 is the initiator molecule for the intracellular transcription of TGF-β. Progressive increase in expression of SMAD 2 accentuates the role of SMAD signalling pathway in fibro genesis. However the intact SMAD signalling pathway is to be studied for appropriate validation and derivation of therapeutic targets. 
